# Incidence and Risk Factors for Severe Dehydration in Hospitalized Children in Ujjain, India

**DOI:** 10.3390/ijerph17020616

**Published:** 2020-01-18

**Authors:** Abhishek Sharma, Aditya Mathur, Cecilia Stålsby Lundborg, Ashish Pathak

**Affiliations:** 1 Department of Pediatrics, Gardi Medical College, Ujjain 456006, India; abhisharma82@gmail.com (A.S.); dr.adityamathur121@gmail.com (A.M.); 2 Global Health-Health Systems and Policy: Medicines, Focusing Antibiotics, Department of Global Public Health, Karolinska Institutet, 171 77 Stockholm, Sweden; cecilia.stalsby.lundborg@ki.se; 3 Department of Women and Children’s Health, International Maternal and Child Health Unit, Uppsala University, 753 27 Uppsala, Sweden

**Keywords:** diarrhoea, severity, severe dehydration, children, risk factors, Ujjain, India

## Abstract

Diarrhoea contributes significantly to the under-five childhood morbidity and mortality worldwide. This cross-sectional study was carried out in a tertiary care hospital in Ujjain, India from July 2015 to June 2016. Consecutive children aged 1 month to 12 years having “some dehydration” and “dehydration” according to World Health Organization classification were eligible to be included in the study. Other signs and symptoms used to assess severe dehydration were capillary refill time, urine output, and abnormal respiratory pattern. A questionnaire was administered to identify risk factors for severe dehydration, which was the primary outcome. Multivariate logistic regression modeling was used to detect independent risk factors for severe dehydration. The study included 332 children, with mean ± standard deviation age of 25.62 ± 31.85 months; out of which, 70% (95% confidence interval [CI] 65 to 75) were diagnosed to have severe dehydration. The independent risk factors for severe dehydration were: child not exclusive breastfed in the first six months of life (AOR 5.67, 95%CI 2.51 to 12.78; *p* < 0.001), history of not receiving oral rehydration solution before hospitalization (AOR 1.34, 95%CI 1.01 to 1.78; *p* = 0.038), history of not receiving oral zinc before hospitalization (AOR 2.66, 95%CI 1.68 to 4.21; *p* < 0.001) and living in overcrowded conditions (AOR 5.52, 95%CI 2.19 to 13.93; *p* < 0.001). The study identified many risk factors associated with severe childhood dehydration; many of them are modifiable though known and effective public health interventions.

## 1. Introduction

Diarrhoea is the second leading cause of morbidity and mortality among under-5 (U-5) children worldwide [1,2,3]. Childhood diarrhoea results in the death of approximately, 700,000 U-5 children yearly, constituting almost 16% of global child death [2,3]. Apart from deaths, the grave consequence of diarrhoea in the first two years of life is its effect on growth, leading to stunting [4]. The morbidity of childhood diarrhoea is about 3 episodes per child per year, and childhood diarrhoea is concentrated in Southeast Asia and Sub Saharan Africa [1]. Controlling diarrhoeal diseases has been on the public health agenda since long. The World Health Organization (WHO)-led diarrhoeal disease control programme resulted in a steep reduction of 75% in mortality due to diarrhoea worldwide from the 1980s to 2008 [5]. However, since then, gains in the reducing mortality rates have been levelling [5]. In 2013, the WHO and the United Nations Children’s Fund (UNICEF) formulated the integrated Global Action Plan for Pneumonia and Diarrhoea (GAPPD), which outlines a framework for ending preventable child deaths due to diarrhoea and pneumonia by 2025 [1]. The GAPPD emphasises a ‘protect, prevent, and treat’ approach that integrates interventions with proven effectiveness [1].

In India, steady progress has been made in reducing deaths in U-5 children, with total deaths declining from 2.5 million in 2001 to 1.5 million in 2012, with a mean rate of fall of around 3.7% annually [1,6]. Despite this reduction, in India, diarrhoea is the third most common cause of death in U-5 children and is responsible for 13% of deaths in this age group [2,7]. Thus, in India alone diarrhoea results in the death of an estimated 300,000 children each year [2,7]. In terms of diarrhoeal episodes per year, the morbidity data from the National Family Health Survey-4 (NFHS-4) in Madhya Pradesh (M.P.) province where the current study was conducted, found that, 9.5% of all U-5 children had suffered from diarrhoea in the 2 weeks prior to survey [8]. The province of M.P. has the highest burden of infant and childhood mortality in India [8].

Despite its consequences on health and survival, the last decade has witnessed a reduction in research on childhood diarrhoea [5]. As an example, only a few studies on epidemiological and clinical risk factors in childhood diarrhoea from India, have been conducted [9,10,11,12,13], but the burden of diarrhoeal diseases remains high [14]. To achieve the goal of ending preventable deaths due to diarrhoea the WHO and UNICEF emphasise the need for more research focusing on identifying context-specific risk factors and interventions to control childhood diarrhoea [1]. Thus, the present study aimed to examine the demographic, socioeconomic, environmental, and clinical risk factors for diarrhoea in children aged less than 12 years in the city of Ujjain, M.P., India.

## 2. Materials and Methods

This prospective observational study was conducted from July 2015 to June 2016. This study was approved by the Institutional Ethics Committee (IEC) of RD Gardi Medical College, Ujjain (IEC reference number 459/2014).

### 2.1. Study Setting

The study was done in the pediatric ward of C.R. Gardi Hospital (CRGH), Ujjain. The CRGH is situated approximately six kilometers from Ujjain city and is a teaching hospital attached to R.D. Gardi Hospital (RDGMC). Department of Pediatrics in RDGMC has 90 beds out of the total 800 beds in CRGH. The hospital is managed by a charitable trust.

### 2.2. Study Participants

**Figure 1:**
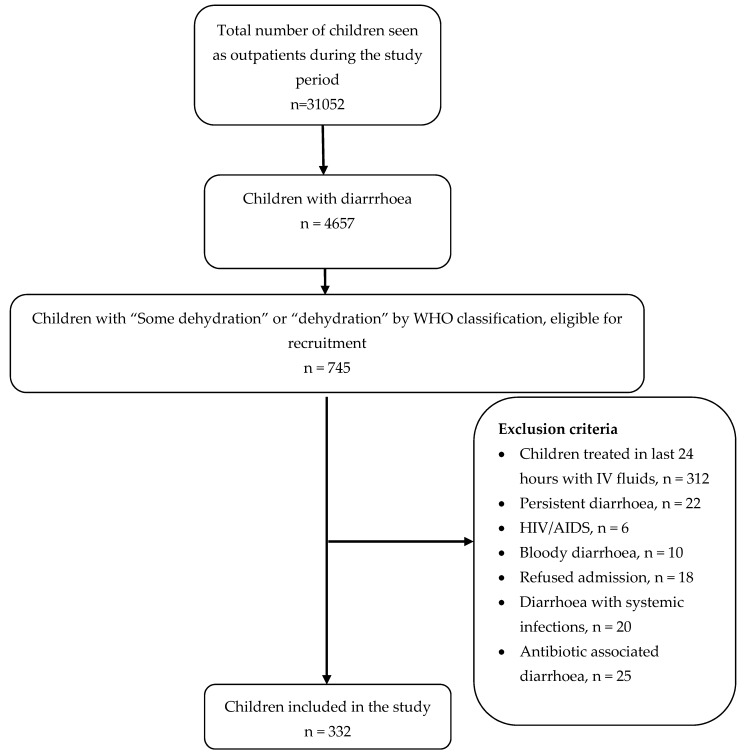
Flow chart of the patient recruitment process.

Consecutive patients aged between 1 month and 12 years who were admitted for acute diarrhoea (up-to 14 days) to the paediatric ward were included in the study. The WHO definition of acute diarrhoea was used: i.e., passage of three or more loose stools (liquid or watery stool) for more than one day [1]. Children having “some dehydration” and “dehydration” according to WHO classification were eligible to be included in the study [15]. Other signs and symptoms used to assess severity of dehydration included capillary refill time, urine output and abnormal respiratory pattern [16]. Children treated in the last 24 h with intravenous fluids, children with persistent diarrhoea (>14 days), children living with human immunodeficiency virus/acquired immune deficiency syndrome, bloody diarrhoea, diarrhoea due to systemic infection, and diarrhoea during a course of antibiotic therapy were excluded from this study. Figure 1 shows the patient recruitment process.

### 2.3. Definitions

The following definitions were used for the present study: nuclear family was defined as a family that consisted of a married couple and their children occupying the same dwelling space [17]. A joint family comprised of more than one married couple and their children who lived together in the same household and shared a common kitchen [17]. Overcrowding was defined as a situation in which more people are living within a single dwelling than there is space for, so that movement is restricted, privacy secluded, hygiene impossible, rest and sleep difficult [17]. Breastfeeding was considered exclusive if the infant has received only breast milk from his/her mother or a wet nurse, or expressed breast milk, and no other liquids or solids [18]. Severe acute malnutrition (SAM) was assessed and managed in children between the ages of 6 months to 5 years according to the consensus statement by the Indian Academy of Paediatrics (IAP) [19]. A *kutcha* house was one with the walls and/or roof made of material such as un-burnt bricks, bamboos, mud, grass, reeds, thatch, loosely packed stones, etc. [20]. A *pucca* house is one, which has walls and roof made of burnt bricks/stones packed with lime or cement [20].

### 2.4. Data Collection Method

The mother or caregiver accompanying the child fulfilling the inclusion criteria was interviewed by one of the research assistants using a predefined questionnaire (online supplementary data). Information was collected on demographics of the child and mother, relevant medical history, history of treatment received before hospitalisation, and environmental and personal hygiene-related risk factors for diarrhoea. Signs and symptoms of children included in the study was recorded at time of admission. Apart from the first author, two independent paediatric consultants assessed each admitted child and decided the management of the child.

### 2.5. Clinical Management of Diarrhoea

During the hospital stay, fluid management of all children were managed according to the IAP protocol for management of diarrhoea [21]. All children received Oral Rehydration Solution (ORS) and oral zinc as soon as oral intake was established [21].

### 2.6. Data Management and Statistical Analysis

Data were entered into EpiData Entry (Version 3.1, EpiData Software Association, Odense, Denmark) and were analysed using Stata (Version 13.0, Statacorp. Texas, TX, USA). The Pearson chi-square test was used to evaluate the association of each risk factor with severe dehydration, and the results are reported as unadjusted odd ratios (OR). Stepwise multivariate logistic regression models, with backward elimination of predictor variables having a p value of more than 0.1 were used to develop the final model. In the final model, the p value of all predictor variables was less than 0.1, except for age and sex. Adjusted OR (AOR) and their 95% confidence intervals (CI) were then calculated. A *p* value of <0.05 was considered significant. Model discrimination was conducted using the C-statistics-receiver-operating-characteristics (ROC) curve, and model calibration was performed using Hosmer–Lemeshow ‘goodness-of-fit’ test [22].

## 3. Results

During the study period, 332 children (54% boys and 46% girls) with diarrhoea were enrolled. The mean age of children was 25.62 months (SD ± 31.85). Of the 332 children admitted with diarrhoea, 232 children were diagnosed to have severe dehydration and the remaining had “some dehydration” according to WHO classification. The prevalence of severe dehydration was thus 70% (95% CI 65 to 75). The signs and symptoms at the time of admission are shown in Table 1. None of the children included in the study died during the hospital stay.

Although slightly more boys were admitted then girls there was no statistically significant difference in the prevalence of severe dehydration among the boys and girls. Most (74%) children belonged to younger age group of 1 month to 24 months. Children of illiterate mothers compared to children of literate mothers and children of working mothers compared to non-working mothers, had increased risk for severe dehydration (Table 2).

Association severe dehydration with feeding-related factors, other factors in past one month-Vitamin A supplementation, measles and diarrhea are shown in Table 3. Presence of SAM, and if the child received vaccination on schedule were also associated with severe h = dehydration. Treatment-related factors like antibiotics, ORS and zinc, and home treatment for the present episode of diarrohea were significantly associated with severe dehydration (Table 3).

The environmental and household sanitation conditions identified as risk factors are shown in Table 4.

### 3.1. Multivariate Analysis

The independent risk factors for severe dehydration were: lack of exclusive breastfeeding in first six months of life, history of measles in the last one month, excessive crying, presence of malnutrition, receiving antibiotic in last 7 days, not receiving oral rehydration solution before hospitalization, not receiving oral Zinc before hospitalization, and living in overcrowded conditions (details in Table 5).

### 3.2. Model Performance

The ROC of the final model was 0.8981 showing excellent model fit as a value more than 0.75 (and near one) predict excellent discrimination [22]. The Hosmer–Lemeshaw test showed that chi-square was 3.94 (*p* = 0.8626). A high p showing good model calibration [22]. 

## 4. Discussion

This is the first study from central India and from the province of Madhya Pradesh to define risk factors for severe dehydration in children with acute watery diarrhoea. Despite measures to control the disease at national level in India the disease burden remains high [14].

In our study, bivariate analysis revealed that children living in urban areas had a lower risk of severe dehydration than children living in rural areas. This may be due to presence of greater number of risk factors associated with diarrhoea among children living in urban areas [23,24]. However, increased risk of acute diarrhoea in rural areas has also been reported [25]. The most important underlying factors in both urban and rural areas are water, sanitation and hygiene (WASH)-related an include the faecal contamination of drinking water, the lack of personal hygiene especially during water handling, and inadequate hand washing after defecation [9,10,26,27]. The children of illiterate mothers have a nearly two-fold increased risk of severe dehydration in our study. Similar increased risk of diarrhoea has been reported in children born to illiterate mothers [5,28].

It has been well documented that the morbidity of diarrhoea is the lowest in exclusively breastfed children, due to the protective effects of breast milk [1,29,30]. Bottle-feeding has been shown to be associated with diarrhoea in India as well as in other low-income countries [24,31]. In the present study, 84% of children received vitamin A supplementation. In our study, the association of vitamin A supplementation with severe dehydration was not statistically significant. However, evidence shows that vitamin A supplementation reduces diarrhoea mortality by 12% and significantly reduces morbidity due to diarrhoea and measles [32]. In our study, bivariate analysis results support that post-measles diarrhoea is an important complication of measles, and children with measles have a 1.29 times increased risk of severe dehydration. Acute diarrhoea has been reported in more than 50% of cases of complicated measles [33]. In our study, SAM was present in 33% of children; of these children, 79% had severe dehydration (*p* < 0.05). The presence of SAM, as defined by IAP’s weight-for-age classification, increases the risk of severe dehydration (Table 5). SAM can increase the severity of diarrhoea, particularly in wasted children, which may be due to the diminished immune response to infection [24]. In our study, according to bivariate analysis, the odds of severe dehydration increased when an antibiotic was prescribed to a child for the present episode of diarrhoea in the last 7 days. Moreover, patients who did not receive ORS and zinc had a higher risk of diarrhoea. Antibiotics are indicated only for a fraction of cases of acute diarrhoea but are prescribed to up to 70% of children with diarrhoea [34]. According to the NFHS-4 data for Ujjain district, 64% and 52% of U-5 children with diarrhoea received ORS in urban and rural areas, respectively. Compared with the NFHS-4 data, the ORS use rates in our study were 32% and 37% in rural and urban areas, respectively [8]. Moreover, according to the NFHS-4 data, 20% of U-5 children received zinc, which is comparable to the rate of 19% reported in our study [8].

In our study, we explored the association of the type of household sanitation with severe dehydration. The absence of a toilet in the house, non-use of toilet, and practice of open-air defecation increased the risk of severe dehydration. In our study, 86% of households had a toilet facility. An increase in the number of toilets constructed at households and the use of toilets can be linked to the Swachh Bharat campaign initiated by the Government of India from 2^nd^ October 2014 onwards [33]. The association of overcrowding with severe dehydration found in our study is probably a proxy for poor sanitation, lack of access to clean water, and inadequate personal hygiene, which are responsible for approximately 88% of childhood diarrhoea in India [24]. Moreover, in many places in India, child faeces are not disposed of safely [10,24,26]. Similar WASH-related environmental risk factors for diarrhoea have been reported recently from Mozambique, Tanzania and Nepal [9,35,36]. Environmental enteric dysfunction is now considered the most important causes of stunting in children with diarrhoea in low-middle-income countries [4,37]. The lack of hand hygiene is a universal risk factor for diarrhoea [38]. It has been estimated that proper hand washing practices can reduce the number of diarrhoeal episodes by more than one-third [38]. However, the best approach to promote hand hygiene among children remains elusive [39].

### Strengths and Limitations

The main strength of this cross-sectional study is that we collected both epidemiological and clinical data for risk assessment of severe dehydration in childhood admitted with acute diarrhoea, which is rare. The study also identified many modifiable risk factors for childhood diarrhoea, which can be used for planning community interventions. However, the study has certain limitations: the study did not ascertain the aetiology of diarrhoeal diseases in children, because it was not the primary study objective and also because of limited laboratory on-site capacity and the lack of affordable point-of-care diagnostics. A home visit to the children with diarrhoea could have helped us identify the contamination of household drinking water, but this was not thought to be feasible.

## 5. Conclusions

Our study identified a multitude of risk factors for childhood diarrhoea, and many of them are modifiable. Promotion of breastfeeding, early identification and treatment of severe acute malnutrition, and treatment of diarrhoea with ORS and zinc in the household and community can reduce the risk for severe dehydration. Research should be conducted to identify effective interventions that can modify the identified risk factors. The implementation of such effective interventions may substantially reduce the morbidity and mortality of diarrhoea in this and similar resource-constrained settings.

## Figures and Tables

**Table 1:** Signs and symptoms of the 332 children admitted with severe dehydration included in the study in Ujjain, India.

Variable	Total (*n* = 332) (%)	Variable	Total (n = 332) (%)
**Eye-ball appearance ^a^**		**Abnormal respiratory pattern ^b^**	
Sunken	270 (81)	Yes	89 (27)
Normal	62 (19)	No	243 (73)
**Ability to drink ^a^**		**Poor oral intake**	
Normal	9 (3)	Yes	291 (88)
Eagerly	300 (90)	No	41 (12)
Poorly	23 (7)	Normal	13 (4)
**Irritability/Restlessness ^a^**		**Sunken anterior fontanelle ^c^**	
Yes	64 (19)	Yes	37 (11)
No	268 (80)	No	295 (89)
**Lethargic/Unconscious ^a^**		**Fever**	
Yes	179 (54)	Yes	289 (87)
No	153 (46)	No	43 (13)
**Skin Pinch ^a^**		**Vomiting**	
Normal	9 (3)	Yes	235 (71)
Slow	300 (90)	No	97 (29)
Very Slow	23 (7)	**Tenesmus**	
**Capillary refill time ^b^**		Yes	95 (29)
Delayed/prolonged	298 (90)	No	237 (71)
Normal	34 (10)		
**Urine output ^b^**			
Decreased	301 (91)		
Normal	31(9)		

a-WHO classification (Reference [15]); b-Other signs and symptoms used for classifying severe dehydration (Reference [16]); c-assessed in children till one year of age.

**Table 2:** Association of socio-demographic and birth-related risk factors with severe dehydration in 332 children hospitalized with diarrhoea.

Variable	Severe Dehydration						
	Total (%) ^a^ (*n* = 332)	Yes (%) ^b^ (*n* = 232)	No (%) ^b^ (*n* = 100)	OR	95% CI		*p* Value
					Lower	Upper	
**Gender**							
Boys	179 (54)	123 (69)	56 (31)	R			
Girls	153 (46)	109 (71)	44 (29)	1.03	0.90	1.19	0.616
**Age**							
1 to 24 months	246 (74)	177 (72)	69 (28)	R			
>2yr to 12yr	86 (26)	55 (64)	31 (36)	0.88	0.74	1.06	0.792
**Resident**							
Urban	243 (73)	160 (66)	83 (34)	R			
Rural	89 (27)	72 (81)	17 (19)	1.22	1.07	1.40	0.003
**Prematurity**							
Yes	321 (97)	224 (70)	97 (30)	R			
No	11 (3)	8 (73)	3 (27)	1.04	0.72	1.50	0.826
**Term low birth weight**							
Yes	45 (14)	28 (62)	17 (38)	R			
No	287 (86)	204 (71)	83 (29)	1.14	0.89	1.45	0.276
**Mother’s education**							
Literate	252 (76)	167 (66)	85 (34)	R			
Illiterate	80 (24)	65 (81)	15 (19)	1.22	1.06	1.40	0.004
**Mother’s occupation**							
Housewife	281 (85)	190 (68)	91 (32)	R			
Working	51 (15)	42 (82)	9 (18)	1.21	1.04	1.41	0.010

a = column percentage, b = row percentage, R-reference

**Table 3:** Association of feeding, other past history and past treatment-related risk factors with severe dehydration in 332 hospitalized children.

Variable	Severe Dehydration						
	Total (%)^a^ (*n* = 332)	Yes (%)^b^ (*n* = 232)	No (%)^b^ (*n* = 100)	OR	95%CI		*p* Value
					Lower	Upper	
**Exclusive breastfeeding (first 6 months)**							
Yes	228 (69)	143 (63)	85 (37)	R			
No	104 (31)	89 (86)	15 (14)	1.36	1.20	1.54	<0.001
**Bottle feeding (at present)**							
No	253 (76)	169 (67)	84 (33)	R			
Yes	79 (24)	63 (80)	16 (20)	1.91	1.03	1.37	0.014
**Vitamin A supplementation (past 1 month)**							
Yes	280 (84)	195 (70)	85 (30)	R			
No	52 (16)	37 (71)	15 (29)	1.02	0.84	1.23	0.824
**History of measles (past 1 month)**							
No	299 (90)	203 (68)	96 (32)	R			
Yes	33 (10)	29 (88)	4 (12)	1.29	1.11	1.50	0.001
**History of diarrhoea (past 1 month)**							
No	140 (42)	88 (63)	52 (37)	R			
Yes	19 2(58)	144 (75)	48 (25)	1.19	1.02	1.38	0.022
**SAM (diagnosed on admission)**							
No	221 (67)	144 (65)	77 (35)	R			
Yes	111 (33)	88 (79)	23 (21)	1.21	1.06	1.39	0.005
**Vaccination on schedule**							
Yes	316 (95)	218 (69)	98 (31)	R			
No	16 (5)	14 (88)	2 (12)	1.26	1.03	1.54	0.019
**Antibiotic received for present episode**							
No	209 (67)	123 (59)	86 (41)	R			
Yes	123 (37)	109 (89)	14 (11)	1.50	1.32	1.71	<0.001
**ORS received for present episode**							
Yes	111 (33)	98 (88)	13 (12)	R			
No	221 (67)	134 (61)	87 (39)	1.47	1.29	1.66	<0.001
**Oral Zinc received for present episode**							
Yes	64 (19)	21 (33)	43 (67)	R			
No	268 (81)	211 (79)	57 (21)	2.23	1.68	3.42	<0.001
**Home treatment given for present episode**							
Yes	67 (20)	58 (87)	9 (13)	R			
No	265 (80)	174 (66)	91 (34)	1.31	1.15	1.49	<0.001

a = column percentage, b = row percentage, SAM = Severe acute malnutrition, ORS = Oral rehydration solution.

**Table 4:** Association of environmental and personal hygiene-related risk factors with presence or absence of severe dehydration in 332 hospitalized children.

Variables	Severe Dehydration						
	Total (%) ^a^ (*n* = 332)	Yes (%) ^b^ (*n* = 232)	No (%) ^b^ (*n* = 100)	OR	95% CI		*p* Value
					Lower	Upper	
**Type of family**							
Nuclear	147 (44)	106 (72)	41 (28)	R			
Joint	185 (56)	126 (68)	59 (32)	0.94	0.82	1.08	0.427
**Type of home**							
*Pacca*	235 (71)	157 (67)	78 (33)	R			
*Kutcha*	97 (29)	75 (77)	22 (23)	1.15	1.00	1.33	0.042
**Over-crowding**							
No	50 (15)	27 (54)	23 (46)	R			
Yes	282 (85)	205 (73)	77 (27)	1.34	1.30	1.75	0.028
**Toilet present**							
Yes	285 (86)	193 (68)	92 (32)	R			
No	46 (14)	38 (82)	8 (18)	1.21	1.04	1.42	0.012
**Use of toilet**							
Yes	283 (85)	191 (67)	92 (33)	R			
No	49 (15)	41 (83)	8 (17)	1.23	1.06	1.43	0.004
**Open air defecation**							
No	284 (86)	192 (68)	92 (32)	R			
Yes	47 (14)	39 (83)	8 (17)	1.22	1.05	1.42	0.008
**Hand washing after going to toilet**							
Yes	298 (90)	202 (68)	86 (32)	R			
No	34 (10)	30 (88)	4 (12)	1.30	1.12	1.50	<0.001
**Finger nail trimmed**							
Yes	277 (83)	188 (68)	89 (32)	R			
No	55 (17)	44 (80)	11 (20)	1.71	1.00	1.37	0.038
**Eating open/stale food**							
No	161 (49)	114 (71)	47 (29)	R			
Yes	171 (51)	118 (69)	53 (31)	0.97	0.84	1.12	0.720

a = column percentage, b = row percentage.

**Table 5:** Multivariate analyses of socio-demographic, past treatment-related, environmental and sign and symptom-related risk factors for severe dehydration in 332 children hospitalized with diarrhoea.

Variable	Adjusted OR	95% CI		*p* Value
		Lower	Upper	
Age in months * (continuous variable)	1.26	0.65	2.46	0.483
Sex * (boys vs. girls)	0.99	0.98	1.01	0.980
Lack of exclusive breastfeeding in first 6 months of life	5.67	2.51	12.78	<0.001
SAM (yes vs. no)	2.05	1.10	5.32	0.027
History of not receiving ORS before hospitalization	1.34	1.01	1.78	0.038
History of not receiving oral zinc	2.66	1.68	4.21	<0.001
Living in overcrowded house (yes vs. no)	5.52	2.19	13.93	<0.001

* Adjusted for age and sex, SAM = Severe Acute Malnutrition, ORS = Oral rehydration solution.
